# The Effect of Calcium Hydroxide on the Short and Long-Term Sealing Properties of MTA Apical Barrier

**Published:** 2011-02-15

**Authors:** Maryam Bidar, Reza Disfani, Salman Gharagozloo, Majid Akbari, Armita Rouhani

**Affiliations:** 1. Department of Endodontics, Dental Research Center/Dental School, Mashad University of Medical Sciences, Mashad, Iran.; 2. Department of Endodontics, Dental School, Mashad University of Medical Sciences, Mashad, Iran.; 3. Department of Endodontics, Dental School, Zahedan University of Medical Sciences, Zahedan, Iran.; 4. Department of Restorative Dentistry, Dental School, Mashad University of Medical Sciences, Mashad, Iran.

**Keywords:** Calcium Hydroxide, Dental Leakage, Fluid Filtration, Mineral Trioxide Aggregate

## Abstract

**INTRODUCTION:**

The aim of this study was to evaluate the effects of remnant root canal medicament, calcium hydroxide on the short and long term sealing ability of mineral trioxide aggregate (MTA) apical barrier.

**MATERIALS AND METHODS:**

Fifty single-rooted teeth were prepared and apical resorptions were made using sulfuric acid. The teeth were allocated into two experimental groups and two control groups. In group 1, calcium hydroxide was placed into canals for one week. In group 2, no medication was placed. In both groups, a 4-mm MTA plug was placed in the root canals. The remaining portion of the canal was filled with gutta-percha/sealer. The microleakage was evaluated after 7 days and 3 months using fluid filtration technique. Repeated measurement ANOVA was used for statistical analysis.

**RESULTS:**

There was no significant difference in sealing ability between the two groups in either time periods. In both groups, microleakage decreased after three months but this decrease was not statistically significant.

**CONCLUSION:**

Medication with calcium hydroxide had no adverse effect on the short and long-term sealing properties of MTA plug.

## INTRODUCTION

Root canal treatment of immature teeth entails different endodontic and restorative complications. The absence of apical stop or apical constriction makes it difficult to achieve complete debridement and favorable root canal obturation. In addition, thin root canal walls may fracture under masticatory forces.

Treatment of such teeth is a challenge for both dentist and patient. Apexification is the most popular and commonly used treatment of open apex teeth. This treatment has been defined as a method to induce a calcified barrier in a root with an open apex or the continued apical development of an incomplete root in teeth with necrotic pulp (American Association of Endodontists 2003). This treatment has been traditionally performed using calcium hydroxide [1][2][3][4].

Artificial apical barriers with a variety of materials have been suggested as alternatives to traditional calcium hydroxide apexification [1][2][3][4][5]. Mineral trioxide aggregate (MTA) has become the material of choice in this regard [6]. Popularity of MTA as an artificial apical barrier can be attributed to several factors. For instance, it can be used and placed in the root canal within one visit, thus eliminating the long working time required for calcium hydroxide apexification [7]. MTA is a biocompatible material [8][9][10] which can induce the formation of hard tissue [11][12], and has good sealing properties[13][14].

Interim medicaments such as calcium hydroxide are not easily removed during final apical obturation with MTA. Therefore it would seem necessary to clarify the effects of the remaining calcium hydroxide on the sealing ability of MTA as the apical barrier. The purpose of this study was to evaluate the effects of calcium hydroxide remnants on the short and long term sealing ability of MTA apical barrier.

## MATERIALS AND METHODS

Fifty extracted human maxillary single-rooted teeth were collected. After extraction, the teeth were placed in sterile saline solution and then placed in 5.25% sodium hypochlorite (NaOCl) for five hours. They were then rinsed and again stored in saline solution. The teeth were radiographed and examined for fracture, and internal/external resorption. Clinical crowns were sectioned at the cement-enamel junction with a high-speed diamond saw (D and Z, Darmstadt, Germany) under copious water spray to create a standardized root length of 14mm.

The canals were instrumented using K-files (Dentsply Maillefer, Tulsa, OK, USA) up to master apical file #40 and Gates-Glidden drills #1-4 (Dentsply, Maillefer, Tulsa, OK) in a step back manner. The access opening was sealed with Coltosol (Coltene, Altstatten, Switzerland). In order to develop apical resorption, the roots were submerged in melted rose wax (Cavex Holland, Netherlands) up to 3mm from the apex; subsequently waxed teeth were soaked in 20% sulfuric acid for four days. Subsequently, the teeth were rinsed with a saline solution and the wax was removed with a scalpel (Supa, Tehran, Iran). The temporary filling was also removed from the access opening [15].

The teeth were then randomly divided into two experimental groups (n=20) and two control groups (n=5). The apices of teeth were inserted into a box filled with wet foam to simulate spongy bone. In group 1 (n=20), pure calcium hydroxide mixed with distilled water (Cina Bartar, Tehran, Iran) was placed into the root canals using lentulo spiral (Moyco Union Brach, York, PA) for one week. A radiograph was taken to ensure complete coverage of the canal. The medicament was removed with stainless steel hand files (Dentsply, Maillefer, Tulsa, USA) and 0.5% NaOCl irrigation. In group 2 (n=20), inter canal medicament was not used. In both experimental groups, a 4-mm apical barrier of MTA (ProRoot MTA; Dentsply, Tulsa, USA) was placed into the canals. The MTA was mixed according to manufacturer and a messing gun was used to place the material as close as possible to the apex. A hand condenser was used to condense the material into the apex.

Radiographs were taken to ensure proper placement and thickness of the MTA plug. Moistened paper points were placed in the canals and all specimens were stored at 37°C and 100% humidity for seven days. MTA was then tested to assure an adequate set. The remaining portion of the canal was obturated with warm gutta-percha and a coronal seal was achieved by 2-3 thicknesses of Coltosol (Coltene, Altstatten, Switzerland). Ten teeth served as positive and negative control. Root canals in positive control group were not filled. In the negative control group (n=5), the teeth were filled as in group 2 and all root surfaces and the MTA apical barrier were covered with two layers of nail varnish.

Microleakage was measured by fluid filtration technique at two times [16][17]. The fluid filtration system described by Moradi et al. was used in the present study [18]. In this study, all the specimens were connected to fluid filtration system and the microleakage was measured. Then, the teeth were stored at 37°C and 100% humidity for three months and microleakage was measured again. This system calculates bubble movement, and includes two parts:

***Part 1:*** tubes, micropipette, pipes and tooth sample that transfer pressure to the specimen.

***Part 2:*** recorder of fluid transport. In this study, for the first time, a digital camera (Olympus, C765, Japan- 5 mega pixel) and professional software (AutoCAD, 2006) was used to record and measure the amount of bubble displacement.

The camera was adjusted in the macrograph to take precise picture in a short distance. The apical end of the root (excluding apical foramen) was covered by cyanoacrylate glue (Inter Lock Co., Japan) and was inserted in a latex pipe (Guihua Co., China) and then the free end of the pipe was connected to the system. A balance in the system was achieved after thirty seconds and then the first picture of the bubble position in the micropipette was taken. Four subsequent pictures were taken with 2-minute time intervals (2, 4, 6 and 8 minutes after the first picture). The same steps were repeated for the next samples. Samples were then returned to their storage box for the next 3 months; the same steps were repeated for all samples.

**Figure 1:**
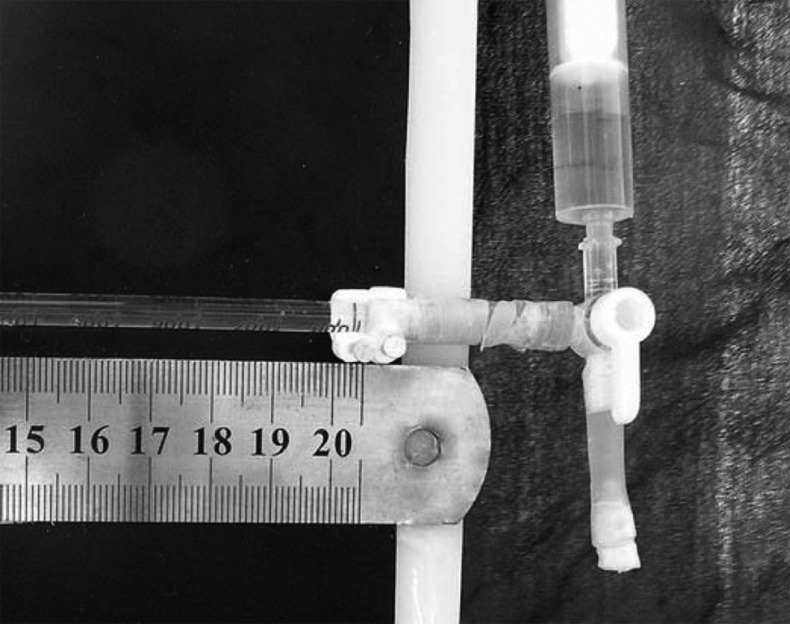
A view of designed fluid filtration system (three-valve tube and its connections)

All pictures (5 for each tooth) were transferred to the computer. The bubble position in each picture was determined by professional software (AutoCAD 2006). These numbers (5 for each sample) were introduced to custom made software designed for accelerating the calculation. This software calculates the mean displacement of the bubble per minute and then with a specific quotient converts the longitudinal displacement of the bubble into the volume of fluid passing from the samples showing it as µL/min/cm H2O. As a result, one number was achieved for each sample that represented the amount of leakage in the root canal as µL/min/cm H2O (Figure 1).

The preliminary analysis with the Kolmogorov Smirnov test was used to confirm normal distribution of the data. The results were analyzed by a repeated measurement ANOVA. In all tests, the significance level was defined as α=0.05.

## RESULTS

The mean microleakage statistics in µL/min/cm H2O are presented in Table 1. After one week and three months, analysis of the results showed that there was no significant difference between group 1 (with calcium hydroxide medication) and group 2 (without calcium hydroxide medication) (P>0.05). In both study groups, microleakage decreased after three months but this decrease was not statistically significant (P>0.05; power=80%).

**Table 1:** The mean (±SD) microleakage in study groups at the two intervals (µL/min/cm H2O)

**Group**	**N**	**7^th^ day**	**3^th^ month**
**G1**	20	0.05±0.00	0.05±0.00
**G2**	20	0.05±0.00	0.05±0.00

Samples showed no leakage in negative control and excessive immeasurable fluid leakage in positive control.

## DISCUSSION

Before placing of MTA as an apical barrier in open-apex teeth, it has been recommended to medicate the canals with calcium hydroxide for one week, with subsequent removal using NaOCl and instrumentation [19]. The rationale is to enhance disinfection of the root canal system in an open-apex tooth. However complete removal of calcium hydroxide from dentinal walls in these teeth is difficult and the remnants of calcium hydroxide may interfere with the proper seal of MTA apical plug.

In this study, instrumentation and 0.5% NaOCl irrigation was used for removal of calcium hydroxide from root canal walls as utilizing higher concentrations of NaOCl in open apex teeth may cause a hypochlorite accident.

The present research showed that medication with calcium hydroxide had no adverse effect on the sealing ability of the MTA plug. This result coincided with the results of Hachmeister et al. who found that one week calcium hydroxide therapy had no effect on the sealing ability of MTA in 70 days [20]. They concluded that the remnants of calcium hydroxide on the root walls in an open apex do not affect the properties of MTA. However, our findings are in contrast to the findings of Stefopoulos et al. who found that calcium hydroxide pretreatment, adversely affected white MTA sealing ability [21]. They assumed that calcium hydroxide may merely be a mechanical obstacle to MTA adaptation with the root canal walls or chemically react with white MTA. In their study, calcium hydroxide pretreatment had no effect on sealing properties of gray MTA. Another study investigated the effects of calcium hydroxide remnants along the canal walls on the sealing ability of gutta-percha and sealer. They found that a significant decrease in dye leakage in canals medicated with calcium hydroxide. They concluded that the calcium hydroxide reacts to form calcium carbonate, providing an initial decrease in permeability [22].

The present study also showed that after three months, the microleakage of MTA decreased; although this decrease was not statistically significant. The improved apical seal after three months may contribute to the expansion of MTA in a wet environment. De Bruyne et al. found that the leakage of MTA decreased after one month although it showed an increase in the 6th month [23]. Martin et al. also found that the apical seal of MTA plugs improved after four weeks, although the difference between apical leakage in 48 hours and four weeks was not statistically significant [24].

## CONCLUSION

This article presents an in vitro procedure and does not reflect the clinical condition; future studies focusing on the success rate of the MTA plug in vivo would further support this treatment option.
